# Mycorrhizal competition release and microbial dynamics in native and non-native *Tuber melanosporum* habitats

**DOI:** 10.1128/aem.00225-26

**Published:** 2026-04-03

**Authors:** Gian Maria Niccolò Benucci, Sergi Garcia-Barreda, Sergio Sanchez, Pedro Marco, Ana Maria De Miguel, Francois Le Tacon, Giorgio Marozzi, Leonardo Baciarelli Failini, Harry Eslick, Todd F. Elliott, Aurelie Deveau, Claude Murat, Domizia Donnini, Gregory Bonito

**Affiliations:** 1 Department of Plant, Soil, and Microbial Sciences, Michigan State University 3078, East Lansing, Michigan, USA; 2 Departamento de Ciencia Vegetal, Centro de Investigación y Tecnología Agroalimentaria de Aragón (CITA) 73040, Zaragoza, Spain; 3 Instituto Agroalimentario de Aragón – IA2 (CITA-Universidad de Zaragoza) 535556 , Zaragoza, Spain; 4 Facultad de Ciencias Departamento de Biología Ambiental, Universidad de Navarra 16754, Pamplona, Spain; 5 Institut National de Recherche pour l'Agriculture, l'Alimentation et l'Environnement (INRAE) Centre Grand Est Nancy 111099 , Champenoux, France; 6 Department of Agricultural, Food and Environmental Sciences, University of Perugia 9309, , Perugia, Italy; 7 Arbor Carbon, Murdoch University 5673 , Murdoch, Western Australia, Australia; 8 Ecosystem Management, School of Environmental and Rural Science, University of New England 1319 , Armidale, New South Wales, Australia; 9 Université de Lorraine, INRAE, IAM 137665, Nancy, France; The University of Arizona, Tucson, Arizona, USA

**Keywords:** microbiome, *Bradyrhizobium*, Australia, Europe, ITS-16S rDNA, ectomycorrhizae, niche theory

## Abstract

Truffles in the genus *Tuber* (Pezizales) are among the few ectomycorrhizal taxa successfully cultivated worldwide. Australia has recently become one of the top producers of *Tuber melanosporum*, a high-valued black truffle native to Europe. Truffle co-introductions in the Southern Hemisphere with their Northern Hemisphere plant symbionts are hypothesized to benefit from reduced ectomycorrhizal competition and natural enemies. In this study, we tested whether *T. melanosporum* in Australia experiences competition release by sampling soils and truffles across France, Spain, Italy, and Australia. Fungal and prokaryotic communities in truffle plantations were assessed in native (European) and non-native (Australian) habitats through ITS and 16S rDNA amplicon sequencing from soil and truffles. Community composition was primarily structured by site of origin and secondarily by presence of brûlé, vegetation-free area induced by truffle production of plant-growth inhibiting compounds, with significant interactions indicating site-dependent brûlé effects. European soils showed higher fungal richness outside the brûlé, with higher evenness overall and for ectomycorrhizal fungi only outside brûlé. *T. melanosporum* showed higher abundance in Australia, with significant differences restricted to outside the brûlé. Overall, ectomycorrhizal fungi in European soils had more than four times the taxa and higher diversity compared to Australian soils. Among the main competitors, *Tomentella*, *Inocybe*, and *Trichophaea* co-dominated in Europe, versus *Scleroderma*, *Hebeloma*, and *Tarzetta* in Australia. Despite differences in soil microbiomes, bacterial communities within *T. melanosporum* truffle ascocarps were strikingly similar across sites and continents and were dominated by *Bradyrhizobium*. Despite high site-level variation, our results support the competition release hypothesis, with reduced enemies benefiting *T. melanosporum* colonization outside the brûlé in Australia.

**IMPORTANCE:**

This study provides the first cross-hemisphere analysis of the truffle microbiome, comparing native and non-native soils and truffles from Europe and Australia. We demonstrate that the remarkable success of *Tuber melanosporum* cultivation in Australia is compatible with ecological release from competitors, which favors its development outside the brûlé. At the same time, we reveal striking cross-hemispheric similarities in truffle-associated bacterial communities, consistently dominated by *Bradyrhizobium*. These findings highlight both the novelty and transcontinental relevance of our work, offering new perspectives on fungal ecology and truffle cultivation.

## INTRODUCTION

There are several lineages of ectomycorrhizal fungi that have evolved truffle forms, whereby fungi sequester their meiospores and form belowground ascocarps (1). Among these truffle lineages, *Tuber* is the most reputed. Ascocarps of *Tuber* are unable to forcibly discharge spores from their asci and instead have evolved the ability to biosynthesize unique aromas—possibly with the assistance of their microbiome—that act as a cue for attracting animals including mammals and insects that consume the truffles and disperse their spores (2–4). It is these unique aromas produced by truffles, particularly species belonging to *Tuber*, that give their reputation and value as culinary delicacies (5, 6).

*Tuber* species are estimated to have evolved ~142 million years ago from a common truffle ancestor and subsequently diversified with different hosts across continents in the Northern Hemisphere (7, 8). Of the estimated >20,000 ectomycorrhizal fungal species, truffles are one of the few to be commercially cultivated (9). This is particularly true for *Tuber melanosporum* Vittad., whereby humans have become an important dispersal agent worldwide through agronomic cultivation practices.

*T. melanosporum* is a winter black truffle species that is native to Europe and the first ectomycorrhizal ascomycete to have its genome sequenced (10). In the wild, *T. melanosporum* is known to grow in calcareous or neutral soils in association with at least the ectomycorrhizal tree hosts in the following genera: *Abies*, *Alnus*, *Carpinus*, *Carya*, *Castanea*, *Cedrus*, *Corylus*, *Fagus*, *Picea*, *Pinus*, *Quercus*, and *Tilia* (11). *T. melanosporum* is highly productive in endemic habitats throughout Southern Europe and particularly in Spain, France, and Italy. Naturally productive sites across Europe show a geographically defined population structure for *T. melanosporum* (12). The first attempts to cultivate *T. melanosporum* are documented in the 18th century in France, and then in the second half of the 19th century, truffle cultivation techniques in southeast and southwest France allowed an increase in production estimated at nearly 1,500 tons (13–15). To overcome the decrease in production occurring in the 20th century, new methods for cultivating *T. melanosporum* were developed in the 1960s and 1970s in France and Italy (16, 17). These approaches are based on ectomycorrhizal synthesis of seedlings with spores and have been demonstrated to be successful for establishing productive truffle plantations in native and non-native soils (6, 17). Consequently, *T. melanosporum* is now successfully cultivated throughout its endemic range in Europe, including Spain, France, and Italy. Truffle production levels vary from year to year, but between 2013 and 2018 averaged ~47 tons/year in Spain, ~43 tons/year in France, and ~19 tons/year in Italy (18).

The first successful fruiting of *T. melanosporum* outside of its native range was in 1987 in California, USA (19). In recent decades, *T. melanosporum* has also been cultivated in many countries in the Southern Hemisphere where *Tuber* and major host plant lineages are not native. For example, successful *T. melanosporum* production has been reported for New Zealand (1993), Australia (1999), Argentina (2016), Chile (2009), and South Africa (2015) (20–23). Outside of its European native range, Australia is now the leading *T. melanosporum* truffle producing country with ~11 tons/year during the time of this study (18).

While still an economically risky long-term enterprise, cultivating *T. melanosporum* can be viable if farmers are able to generate stable production. Planting seedlings well colonized with *T. melanosporum* is a prerequisite for truffle production. This is particularly important when planting truffles outside of their native range given the lack of autochthonous inoculum in these regions. Appropriate soils and a favorable climate and irrigation are also key elements for reliable truffle production (24). Optimal conditions for *T. melanosporum* mycelium development include well-drained, calcareous soils with high pH (typically 7.5–8.5). Adequate moisture through natural precipitation or supplemental irrigation is also critical during growth periods that support fruiting body formation, while Mediterranean-type climates with distinct seasonal patterns encourage proper truffle maturation.

Established productive truffle orchards can be profitable given high yields and market prices for fresh truffles that typically exceed $500/kg. European truffle orchard productivity varies widely, with yields above 50 kg/hectare reported in both mature and recent plantations representing the higher end of production ranges (25). In the United States, yields average 17 kg/hectare but are up to 43 kg/hectare (24). In Australia, there are many orchards with >30 kg/hectare, and remarkably, yields of over 1,000kg/hectare have been reported (*H. Eslick personal communication*). Further, Australia holds the record for the largest cultivated truffle on record (1,511 g), produced by Stuart Dunbar of Yarrow Valley Truffles (*S. Dunbar personal communication*). While generally high production of *T. melanosporum* in Australia could be attributed to agronomic factors such as specific management practices, soil physical/chemical factors, or legacy effects, longer growing seasons in Australia could additionally favor the growth of the host and consequently that of truffle symbionts. The importance of competition release, whereby species introduced into exotic habitats may thrive due to the release from competition and natural enemies and (26) differences in the soil microbial community on a large scale is less clear, and the focus of this work.

Black truffle species are well-known to produce a brûlé around the host tree, defined as an area devoid of vegetation in the rooting zone of the tree host where most truffle ascocarps are produced. The formation of the brûlé is linked to changes in the soil microbial populations and activity, including a decrease in ectomycorrhizal basidiomycete and arbuscular mycorrhizal fungal richness, and changes in the composition of the bacterial community (27–30). The brûlé is also associated with phytotoxic activity of *T. melanosporum*, which reduces plant growth and diversity (31). The characteristics of the brûlé have been found to be related to truffle yields in wild stands (32, 33). Further, it has been demonstrated that truffle fruiting bodies themselves are well-colonized by specific bacterial groups, with some data indicating that the truffle microbiome is important in truffle ecology and its aroma (2, 34–36). Whether microbial communities in brûlés or truffles differ between native and non-native soils remains an open question.

In this study, we test this hypothesis that *T. melanosporum* benefits from competition release in Australian compared to European truffle orchards. Australian ectomycorrhizal forests are dominated by Myrtaceae and Nothofagaceae in Victoria and Tasmania, which have evolved with different ectomycorrhizal fungal symbionts from those of Fagaceae and Betulaceae that are often associated with *T. melanosporum* in the Northern Hemisphere (37–40). Thus, European tree species (e.g., *Quercus robur* L., *Quercus ilex* L., or *Corylus avellana* L.) inoculated with *T. melanosporum* and used as truffle hosts in Australia are expected to experience lower ectomycorrhizal competition pressure. This would result in the dominance of *T. melanosporum* below ground in these orchards, quicker maturity, and greater yields. We also examine how microbial communities in both brûlé soils and truffles differ between their native and non-native geographic ranges. We did this by sampling productive truffle orchard soils, within and outside the brûlés, and truffle fruiting bodies produced in native soils across Europe and non-native soils across Australia. If competition release is evident in Australian truffle orchards, we would expect Australian soils to have lower richness and diversity of ectomycorrhizal fungi and for *T. melanosporum* to be significantly over-represented in the ectomycorrhizal community compared to those of Europe. Further, given that pH is a major driver of bacterial community structure and that orchards are maintained at similar pH (~7.5) (41), we expected that bacterial communities in brûlés would be more similar to outside brûlés rather than other orchards, with continent of origin being the major driver. We further hypothesized that the *T. melanosporum* microbiome inside ascocarps would be fairly conserved between European- and Australian-produced truffles given previous research indicating that fungal taxa are selective in their microbiome (34, 42, 43).

## MATERIALS AND METHODS

### Sampling

Twenty-four truffle sites producing *T. melanosporum* were included in this study: 13 located in Europe (Northern Hemisphere, native to *T. melanosporum*), of which 10 cultivated and 3 natural sites; and 11 located in Australia (Southern Hemisphere, non-native to *T. melanosporum*). Details about the studied sites are provided in Table S1 at https://doi.org/10.6084/m9.figshare.31329013. Each truffle orchard in this study was sampled during the winter (productive) season following the same procedures and sampling design. Briefly, in each orchard, nine individual productive trees growing at least 25 m apart from each other were selected for sampling. Soil inside the brûlé around each tree was sampled 0.5 m from the trunk by first removing all leaf litter and then homogenizing a ~500 cm^3^ block of soil with a four-prong garden cultivator and pooling a teaspoon of homogenized soil from each of the four cardinal points into a single sample. The shovel used for sampling was cleaned with soap water, rinsed two times, and then sterilized with ethanol before every next sample. Soil outside the brûlé was sampled by choosing the furthest point away halfway between the target tree and its nearest neighbor in the next row. In total, 18 samples from each site were collected and analyzed, 9 inside and 9 outside the brûlé in 24 truffle orchards. One to five control outgroup soil samples were taken in a similar manner as described but at least 50 m outside of the orchard in unamended natural forest or pasture lands surrounding the orchard for a total of 90 soils (54 in Australia and 36 in Europe). Orchards in Tasmania, New South Wales, Victoria, and Western Australia were sampled in June 2015. Orchards in Italy, France, and Spain were sampled in January of 2016. Three natural forests producing wild *T. melanosporum* were also sampled in Europe (one in Italy, Spain, and France), as control reference points for the microbial communities in wild truffle habitat. A total of 198 trees were sampled in Australia and 234 trees were sampled in Europe, and with 90 reference control soils under outgroup trees (trees not previously inoculated with truffles), resulted in 522 soil samples for processing. All soils were immediately dried in paper bags containing silica beads and were kept on silica until processing. Soils were analyzed for chemical properties, including pH as well as micro- and macro-nutrients at the Michigan State University soil testing laboratory. For each truffle orchard, two composite samples were created by mixing equal amounts of soil samples from each site: one composite from within brûlé soils and one from outside brûlé soils (48 soil analyses). All other relevant ecological factors, such as, for example, truffle yields and management practices applied and where, were obtained from the orchard owners. In each orchard, we also collected multiple truffles to characterize the internal bacterial microbiome. On average, we obtained approximately 9.5 truffles per site in Australia and 7 truffles per site in Europe, resulting in a total of 104 truffles (19 from 2 Australian sites and 85 from 12 European sites). Truffles were crack-opened with sterile gloves and (under a fume hood, when possible, otherwise on a sanitized surface), a portion of the gleba was sampled as quickly as possible using a sterilized scalpel and put into 2 mL tubes with CTAB buffer (34).

### Molecular methods

DNA was extracted from soil samples with the PowerMag Soil DNA Kit (Qiagen, Carlsbad, CA, USA) following the manufacturer’s instructions, and from truffle gleba with the CTAB buffer-based chloroform extraction protocol as previously described (34). PCR was performed with ∼20 ng of DNA with DreamTaq Green DNA Polymerase (ThermoFisher Scientific, USA). The primer pair ITS1f-ITS4 was used to amplify fungal ITS rDNA from soils, and the primer pair 515F-806R was used to amplify prokaryotic (bacteria + archaea, hereafter bacteria) 16S rDNA from soils and truffle gleba. A protocol modified from Lundberg et al. (44), which employed frameshift primers was used for Illumina library preparation, as described in reference 42. PCR products were visualized through gel electrophoresis via ethidium bromide-stained agarose gels. Samples were normalized using the SequalPrep Normalization Plate Kit (ThermoFisher Scientific) and combined into a single amplicon pool. Amicon Ultra 0.5 mL 50 K filters (EMD Millipore, Germany) were used to concentrate the libraries at about a 20:1 ratio. Primer dimers and other small fragments were removed from libraries performed with Agencourt AMPure XP magnetic beads (Beckman Coulter, USA). Fragment libraries were sequenced on an Illumina MiSeq instrument during 600 cycles with v3 chemistry (Illumina, USA) through the Michigan State University RTSF Sequencing Core.

### Bioinformatics

ITS and 16S rDNA amplicon quality was analyzed with FastQC (45). Sequencing reads were demultiplexed in QIIME based upon sample barcodes (46). Cutadapt was used to trim Illumina adapters and primers from reads prior to further processing (47). Reads were then filtered at a quality threshold and trimmed to the same number of nucleotides (48). Sequences were then de-replicated, singleton sequences excluded, and the remaining sequences were used to create operational taxonomic units (OTUs) at 97% similarity threshold with the UPARSE algorithm (49). Taxonomy was assigned with the RDP Naive Bayesian Classifier (50) using the Greengenes database (51) version gg-13-8 for 16S rRNA gene, and CONSTAX2 (52) based on the UNITE eukaryotic sequence database version 7.1 2016-08-22 (53) ITS rDNA.

### Statistical analyses

Statistical analyses were carried out in R to process OTU tables resulting from bioinformatics, mapping files containing metadata, as well as taxonomy and reference sequences for both marker genes (54). First, OTUs that appeared in the extraction or PCR negative controls (i.e., suspected contaminants) were removed from all samples alongside non-target organisms (42, 55). An ectomycorrhizal data set was constituted by filtering the fungal data set using the *FungalTraits* database and the last taxonomic level we were able to assign to the OTUs (56). Prior to statistical analyses, sequencing data were rarefied to 3,818 sequences per sample for ITS (fungi), 4,437 sequences for the soil 16S (bacteria), 2,000 sequences for the 16S from the truffle gleba, and 1,000 16S sequences for the outgroup soil samples (57).

For both soil samples and truffle gleba samples, *hill_0* (observed richness), *hill_1* (Shannon index), and *hill_2* (invSimpson index) numbers (58) were determined with the *vegan* R package (59, 60) to measure alpha diversity. Fixed effects of continent and brûlé on log(*hill_0*) and log(*hill_1*), with site (i.e., truffle orchard) as random intercept, were analyzed using linear mixed effect models. These models were always retained when compared with models that included the sampled tree nested within the site using Akaike information criterion (AIC) and ANOVA. Diagnostics were generated using the *DHARMa* R package (61) and provided in Fig. S15 at https://doi.org/10.6084/m9.figshare.31329013. Principal coordinate analysis (PCoA) ordinations were generated to assess β-diversity with the “ordinate” functions of the *phyloseq* R package (60). Patterns of diversity were assessed for statistically significant differences between sites and samples in the *vegan* package (59) with the PERMANOVA function “adonis2.” We run the models on the whole data sets, mainly to test the difference in community composition between continents, and then in each continent to more accurately test the effect of site and brûlé, and interactions, on the microbial communities. To assess whether there was a substantial difference from the models run on the Jaccard (based on presence/absence) and Bray-Curtis (based on relative frequencies) distances, we used AIC. Differences in community dispersion were assessed with the “betadisper” function. Due to significant heteroskedasticity between factor levels, we fit multivariate generalized linear models using functions “manyglm” and “anova.manyglm” in the *mvabund* R package (62). Multivariate generalized linear models offer advantages compared to distance-based modeling, such as adonis2, because they control for the confounding mean–variance relationships that affect microbiome data fitting them to a negative binomial distribution (62, 63). We modeled *T. melanosporum* sequence abundance in the soil using a zero-inflated negative binomial mixed model with site as a random intercept estimating the conditional (count) component mean abundance (given *T. melanosporum* presence) and the probability of structural absence, that is, the zero-inflation component. Best models selected as reported above and model diagnostics provided in Fig. S16 at https://doi.org/10.6084/m9.figshare.31329013.

The Random Forest-based feature selection routine, implemented in the *Boruta* R package (64), was run 100 times to identify important features (i.e., OTUs) to predict *T. melanosporum* sequence abundance in the soil. For this analysis, we used *T. melanosporum* sequence abundance as is (i.e., regression) and also the discretized version of it (i.e., classification) that contained three *T. melanosporum* levels: high, medium, and low. An indicator species analysis was used to identify OTUs highly associated with high or low levels of *T. melanosporum* using the “multipatt” function in the *indicspecies* package (65). As we performed analysis on composite samples representing two samples for each truffle orchard, one inside and the other outside the brûlé, we averaged microbiome data to match the chemistry data. A principal component analysis (PCA) was used to visualize and explore patterns and relationships between chemical and microbial properties using the “PCA” function in the *FactoMineR* R package. All variables were rescaled before PCA. Spearman correlation was also used to identify the main significant (*P* ≤ 0.05) trends between chemical and community traits.

## RESULTS

### Sequences and coverage

We analyzed bacterial and fungal microbiomes from 24 orchards in 432 soil samples from productive host trees, 93 reference (i.e., outgroup) control samples outside of truffle orchards, and 104 samples from truffles. The two Illumina MiSeq sequencing of ITS (fungal) and 16S (bacterial) ribosomal DNA resulted in 23,860,471 ITS (from productive and reference soils) and 30,941,017 16S (from productive and outgroup soils, and truffles) demultiplexed reads. Sequences were quality filtered and clustered into 12,830 fungal and 15,980 bacterial OTUs with the UPARSE pipeline. ITS samples were rarefied to 3,818 sequences per sample. Soil 16S samples were rarefied at 4,437 sequences per sample, while 16S samples of truffle gleba and outgroup soil samples were rarefied at 1,000 sequences per sample (statistics and visuals used to set rarefaction depths in Fig. S1 and S2 at https://doi.org/10.6084/m9.figshare.31329013).

### Fungal and bacterial composition of soils

**Fig 1:**
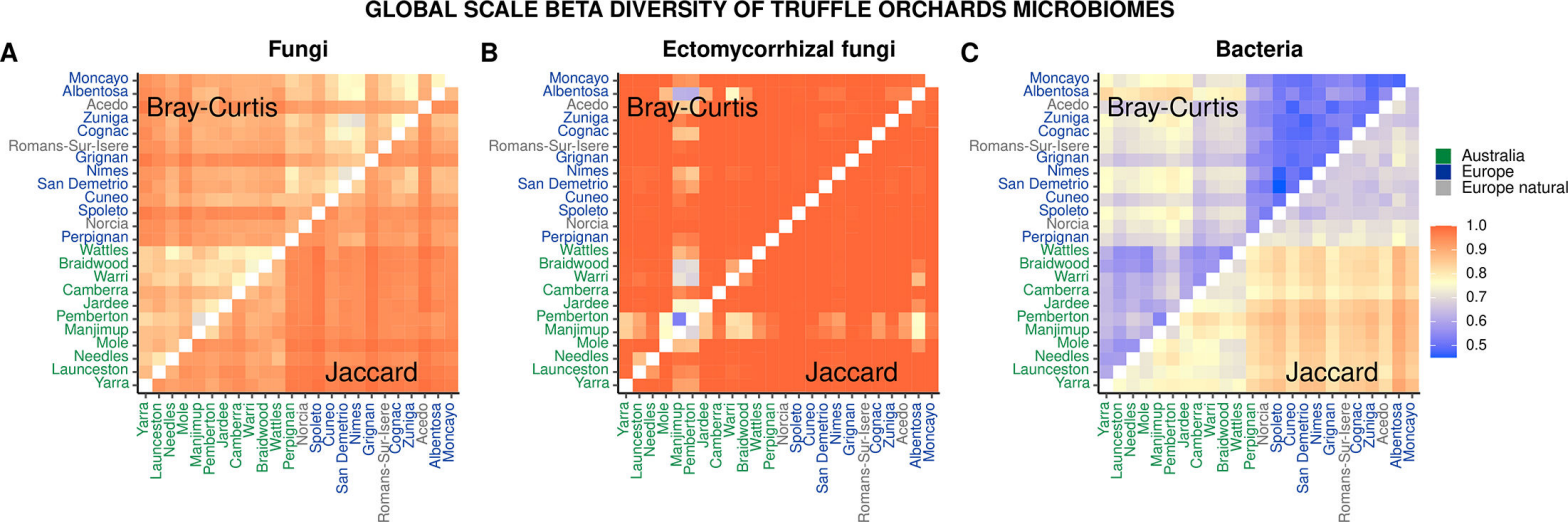
Within truffle orchard median Bray-Curtis and Jaccard distances, both inside and outside the brûlé combined, in the fungal (**A**), ectomycorrhizal fungal (**B**), and bacterial (**C**) data sets.

Median Bray-Curtis dissimilarity distances were statistically (*P* ≤ 0.05) smaller compared to median Jaccard distance between samples for each truffle orchard in all data sets (Fig. 1; also see Fig. S3 at https://doi.org/10.6084/m9.figshare.31329013) and especially in the bacterial data set (Fig. 1C). Both Bray-Curtis dissimilarity and Jaccard median distances were statistically larger (*P* ≤ 0.05) between Australian and European orchards compared to European and European or Australian and Australian orchards (Fig. S3) for fungi (Fig. 1A) and bacteria (Fig. 1C). Large distances were observed for ectomycorrhizal fungi (Fig. 1B), regardless of the continent of origin, except for the two Western Australian orchards of Pemberton and Manjimup, which were more similar to each other.

**Fig 2:**
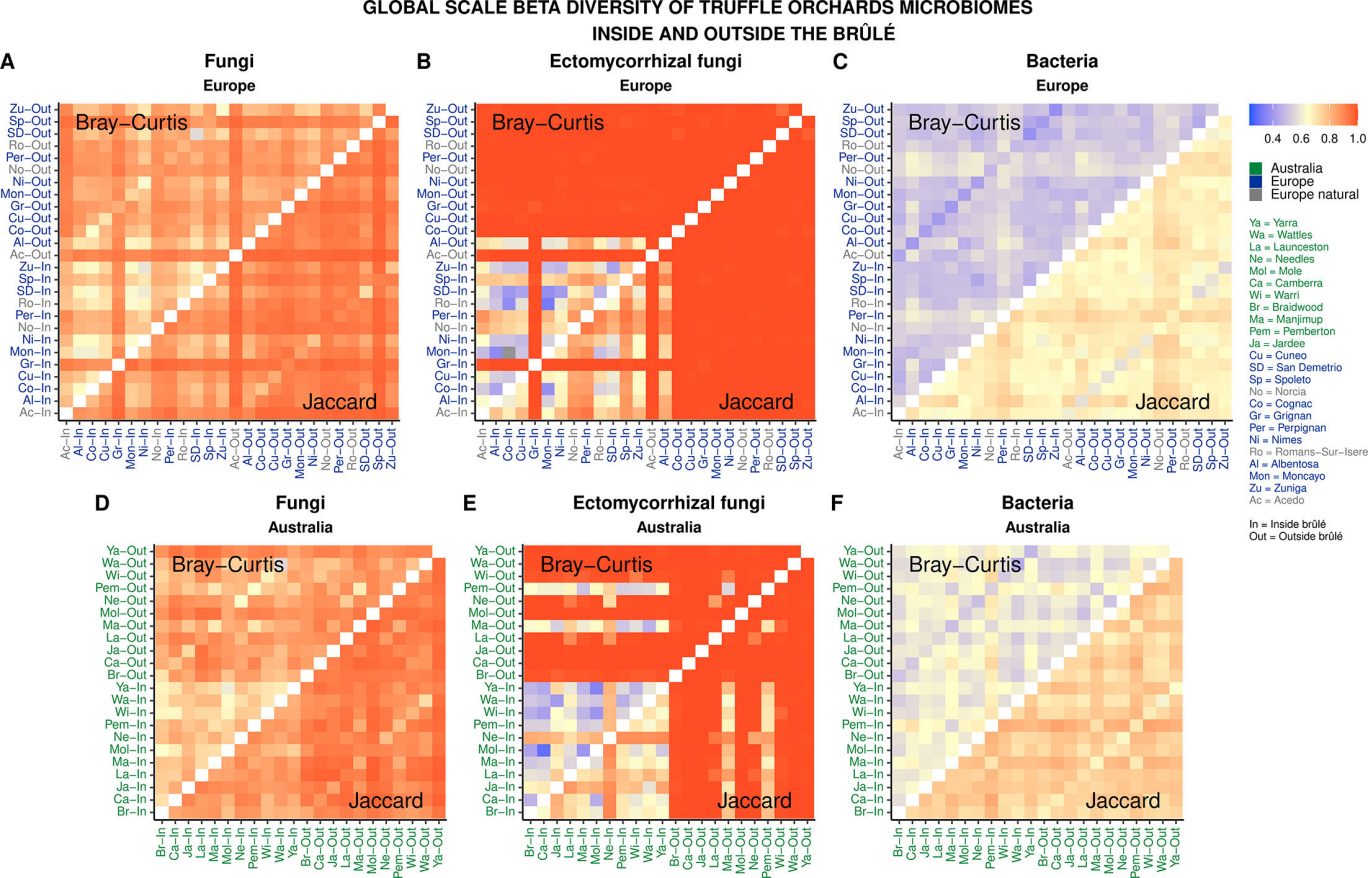
Within truffle orchard median Bray-Curtis Jaccard and distances, separated between inside and outside the brûlé, for European (**A–C**) and Australian (**D–F**) and using the fungal, ectomycorrhizal fungal, and bacterial data sets.

When split by continent of origin (Fig. 2), and median calculated separately for inside and outside the brûlé, both Jaccard and Bray-Curtis median distances were statistically (*P* ≤ 0.05) smaller when samples inside the brûlé were compared to one another, especially evident in the data set of ectomycorrhizal fungi (Fig. 2A through F; also see Fig. S4 and S5 at https://doi.org/10.6084/m9.figshare.31329013) in both European and Australian orchards. Median Bray-Curtis distances were statistically (*P* ≤ 0.05) smaller compared to Jaccard, especially in the bacterial (Fig. 2C and F) data set with a less distinct pattern between inside and outside the brûlé.

Principal coordinate analysis (PCoA) of the fungal, ectomycorrhizal, and bacterial data showed similar trends, but less separation between orchards for ectomycorrhizal fungi and stronger regional separation in Australia between the East coast, the West coast, and Tasmania. In contrast, European sites appeared to be more clustered together, especially for the fungal data sets (see Fig. S6 at https://doi.org/10.6084/m9.figshare.31329013, panels A–C, top). The PCoA for data sets separated by continent (Fig. S6A through C bottom) showed clear clustering of orchards by site and less strongly by being inside or outside the brûlé.

The models resulting from the multivariate analysis of variance (i.e., adonis2) using Jaccard distance showed a better fit (lower AIC and AIC.g) than those based on Bray-Curtis distance.

**Fig 3:**
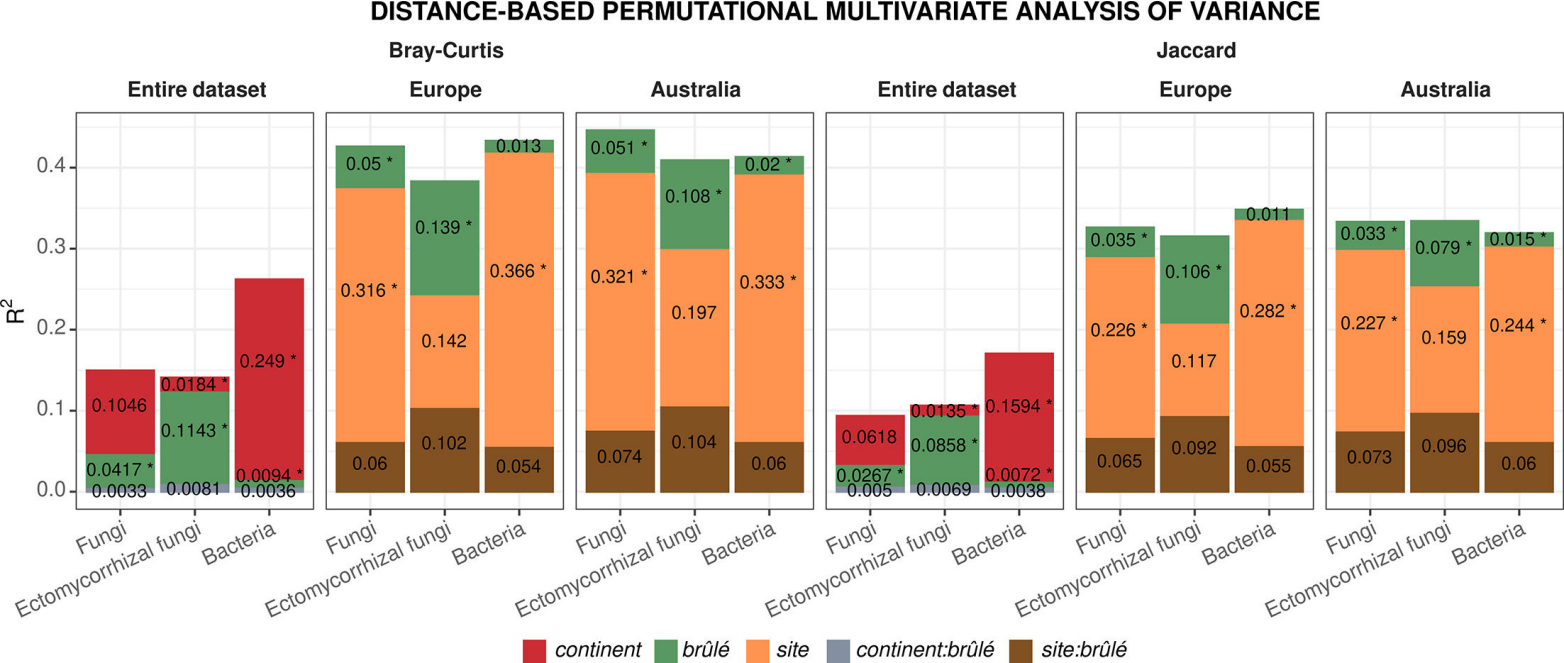
Results from distance-based permutational multivariate analysis of variance (i.e., adonis2) and multivariate homogeneity of groups dispersions (i.e., betadisper) for fungi, ectomycorrhizal fungi, and bacteria using Bray-Curtis and Jaccard distances on the entire data set, as well as, only on the European and Australian sites. For each factor, bar heights show a significant (*P* ≤ 0.05) effect of the factor quantified as *R*^2^. Stars represent significant (*P* ≤ 0.05) differences in multivariate dispersion (i.e., heteroscedasticity) for that factor.

Overall, even with low *R*^2^, significant (*P* ≤ 0.05) interactions between continent and brûlé were detected for all microbial groups besides fungi when Bray-Curtis dissimilarity distance was used, indicating that the difference in microbial structure between Australia and Europe is dependent on the community being inside or outside the brûlé (Fig. 3; also see Table S2 at https://doi.org/10.6084/m9.figshare.31329013). However, the effect of the brûlé was higher for ectomycorrhizal fungi, and the effect of continent was higher for bacteria, compared to those of fungi. Furthermore, we also detected significant variances (i.e., betadisper) between the continent and brûlé levels, which could also lead to significant differences in the detected groups’ averages.

Pairwise adonis2 models comparing sites one another showed significantly (*P* ≤ 0.05) higher *R*^2^ when orchards from Europe were compared to sites from Australia compared to when sites from the same continent are compared one another, and larger effects were detected when using Bray-Curtis (see Fig. S7A through C at https://doi.org/10.6084/m9.figshare.31329013) compared to Jaccard distances (Fig. S7D through F) for fungi, ectomycorrhizal fungi, and bacteria. A few sites were not statistically different in their ectomycorrhizal community structure (Fig. S7B and E). Both site and brûlé, and their interaction, were significant (*P* ≤ 0.05) for fungi, ectomycorrhizal fungi, and bacteria based on both Bray-Curtis and Jaccard (Fig. 3; also see Table S3 at https://doi.org/10.6084/m9.figshare.31329013). The effect of brûlé, and its interaction with the continent, was higher in the ectomycorrhizal fungi compared to all fungi and bacteria. The effect of the brûlé was the smallest in bacterial soil microbiomes. Regarding the dispersion, fungi showed significantly different (*P* ≤ 0.05) dispersion between sites and brûlé levels, while in the ectomycorrhizal fungi, only inside and outside brûlé showed different dispersion, and for the bacteria, only between sites (Fig. 3). Multivariate generalized linear models based on a negative binomial distribution confirmed the presence of significant interactions for fungi, ectomycorrhizal fungi, and bacteria in all models (see Table S4 at https://doi.org/10.6084/m9.figshare.31329013).

### Fungal and bacterial diversity

**Fig 4:**
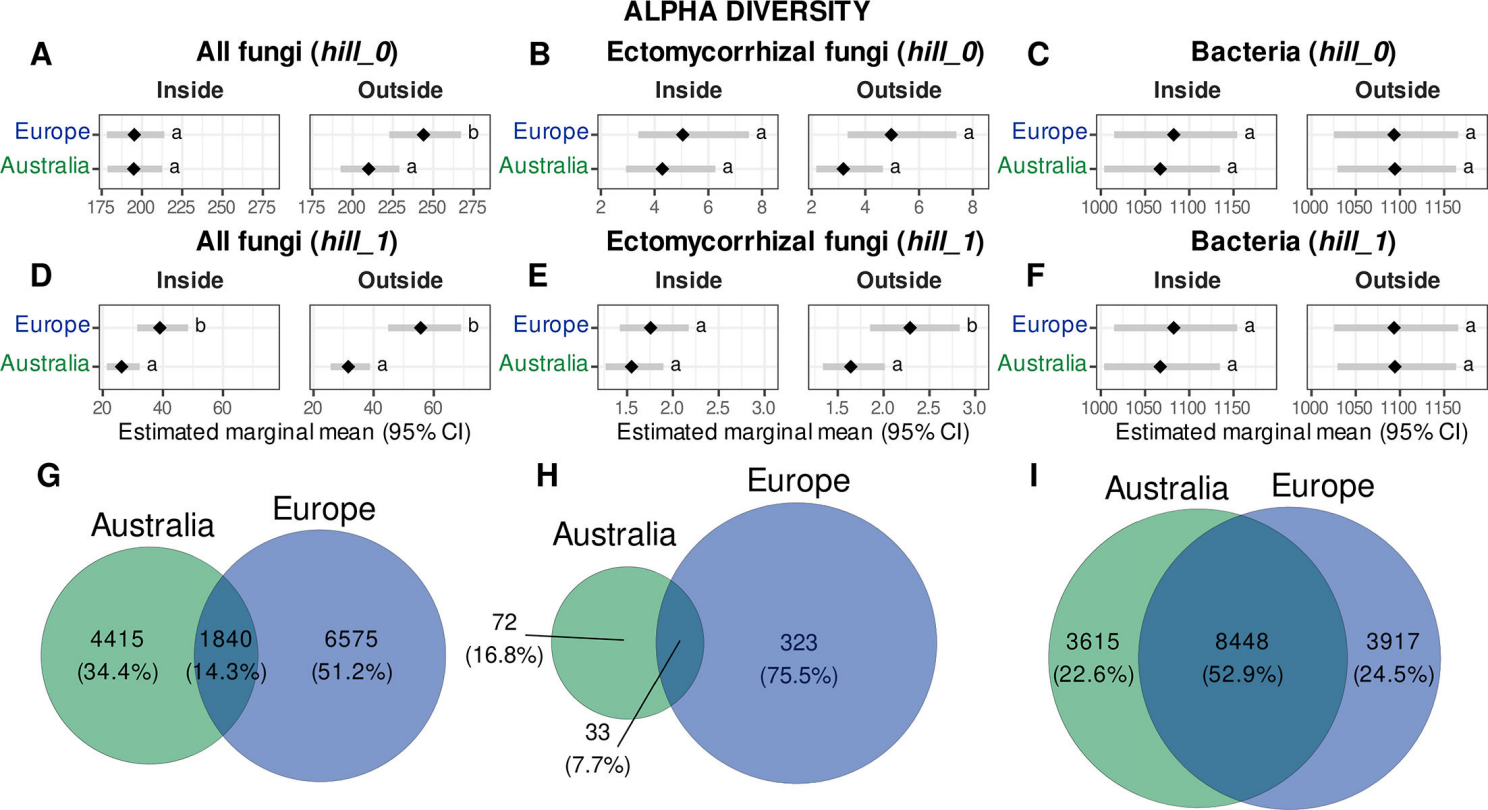
Average *hill_0* (richness) and *hill_1* (Shannon index) between continents and brûlé in the fungi (**A and D**), ectomycorrhizal fungi (**B and E**), and bacteria (**C and F**), respectively. Black diamonds represent estimated marginal means, and gray bars are the 95% CI. Different letters represent significantly different means from the most parsimonious linear mixed effect model for each data set [e.g. log(*hill_0*) ~ continent * brule + (1 | site)] (see Table S5 at https://doi.org/10.6084/m9.figshare.31329013). The total number of fungal, ectomycorrhizal, and bacterial OTUs unique to Australia, Europe, or shared is shown on the Euler diagram (**G, H, and I**), respectively. Estimated means are back transformed to original scale. Natural sites were not included in the analysis.

We examined how alpha diversity metrics responded to truffle cultivation (inside vs outside brûlé) and biogeography (Europe vs Australia) using linear mixed-effect models with site as a random effect (Fig. 4A through F). For fungal richness (i.e., *hill_0*), we observed contrasting patterns between all and ectomycorrhizal fungi. Fungal richness showed a significant continent × brûlé interaction (*t* = 3.13, *P* = 0.002; Fig. 4A), driven by higher richness in European samples collected outside the brûlé (243.9 ± 10.8 SE OTUs) compared to Australian samples (209.9 ± 8.6 SE OTUs; outside brûlé Australia/Europe ratio = 0.861, *P* = 0.021), while inside samples were similar between continents. Ectomycorrhizal fungal richness also showed a significant interaction (*t* = 2.65, *P* = 0.008; Fig. 4B), with a strong main effect of brûlé (lower richness outside; *t* = −3.98, *P* < 0.001), though it did not differ between continents and site-level variation was substantial (site var. = 0.33, residual var. = 0.26). In contrast, bacterial richness showed only a weak main effect of brûlé (*t* = 2.03, *P* = 0.043; Fig. 4C) and no continental differences, remaining approximately stable and between 1,067.2 and 1,094.3 OTUs across treatments (see Table S5 at https://doi.org/10.6084/m9.figshare.31329013).

Shannon diversity patterns (i.e., *hill_1*) differed from richness, suggesting shifts in community evenness. Total fungal Shannon diversity exhibited main effects of both continents (*t* = 2.73, *P* = 0.011) and brûlé (*t* = 2.41, *P* = 0.016; Fig. 4D), with European communities consistently 43.5–76.4% more diverse than Australian communities inside and outside the brûlé (inside brûlé Australia/Europe ratio = 0.674, *P* = 0.012; outside brûlé Australia/Europe ratio = 0.567, *P* = 0.006). Ectomycorrhizal Shannon diversity (Fig. 4E) showed a significant continent × brûlé interaction (*t* = 2.46, *P* = 0.015), with European outside samples significantly more diverse than Australian outside samples (outside Australia/Europe ratio = 0.72, *P* = 0.028), while inside samples were similar. Bacterial diversity mirrored bacterial richness, showing only a main effect of brûlé (*t* = 2.63, *P* = 0.009; Fig. 4F) with no continental differences (Table S5). These results reveal that bacterial communities are remarkably resistant to both truffle cultivation practices and biogeographic factors, while fungal communities, particularly ectomycorrhizal fungi, show complex, context-dependent responses that differ between continents and between the cultivated brûlé zone and surrounding soil.

The Euler diagram showed lower fungal richness in Australia compared to Europe (Fig. 4), particularly for the ectomycorrhizal fungi, of which only about 17% were unique to Australian orchards compared to 75% that were unique to the European orchards. Only 7.6% of the ectomycorrhizal fungal OTUs were shared between the two continents (Fig. 4G and H). On the contrary, bacteria had a higher proportion of shared taxa (nearly 53%), compared to <25% that were unique to either Europe or Australia (Fig. 4I).

When calculated at the orchard level (see Fig. S8 to S11 at https://doi.org/10.6084/m9.figshare.31329013), both richness and inverse Simpson were variable between different sites with a few sites showing significantly higher (*P* ≤ 0.05) values inside the brûlé compared to outside. Several of the European (and Australian) sites showed higher fungal richness outside the brûlé, but the same sites showed higher ectomycorrhizal richness inside the brûlé. Interestingly, no clear pattern of bacterial diversity inside or outside the brûlé was observed.

### Fungal abundance

**Fig 5:**
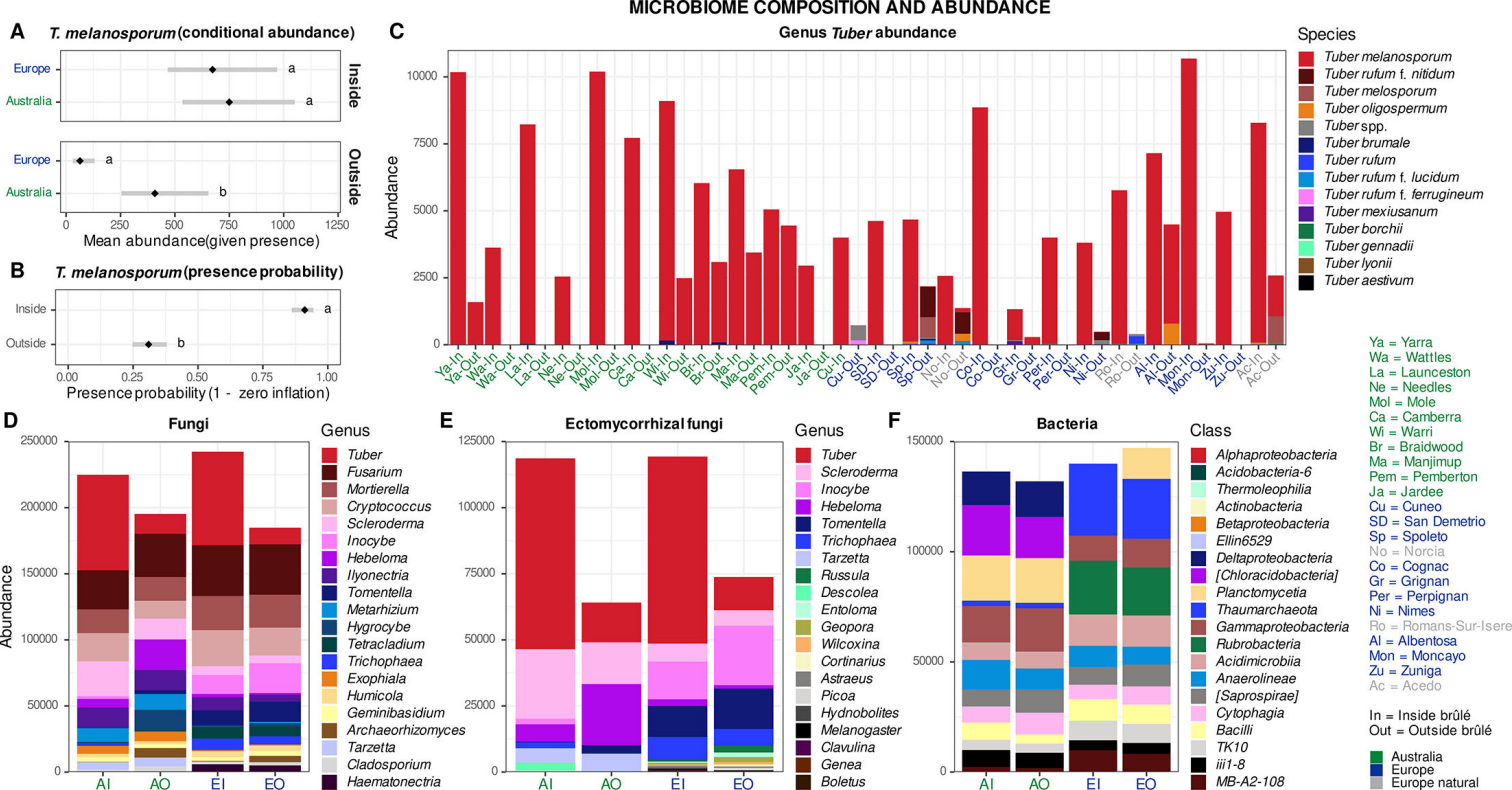
Fungal community composition of the Australian and European truffle orchard. Panels show results from the zero-inflated negative binomial mixed-effects model including continent, brûlé, and their interaction as fixed effects, and orchard (site) as a random intercept. Conditional mean abundance (given presence) showing continent-specific responses to brulé (**A**). Black diamonds and error bars show the model-predicted mean abundance (back transformed to original scale) conditional on presence and 95% CI. Zero-inflation model showing probability of structural absence (**B**). Black diamonds and error bars show model-predicted probability of presence (1 − zero inflation) and 95% CI. Natural sites were not included in any models. (**C**) Sequence abundance of all the *Tuber* species detected in each orchard within and outside the brûlé. (**D and E**) Sequence abundance of the first 20 fungal genera) and (**F**) the first 20 bacterial classes between continents and brûlé. Unclassified taxa were not included.

*T. melanosporum* abundance exhibited strong zero inflation and overdispersion and was therefore analyzed using a zero-inflated negative binomial mixed-effects model with orchards (site) as a random effect. The conditional model (Fig. 5A) indicated that, when present, *T. melanosporum* abundance (conditional on presence) showed a significant continent × brûlé interaction (*z* = −4.00, *P* < 0.001 [see Table S5 at https://doi.org/10.6084/m9.figshare.31329013]). Inside the brûlé, abundance was higher in both Australia (749.8 ± 129.1 SE reads) and Europe (673.2 ± 125.5 SE reads), with no significant difference between continents. However, the effect of sampling location (inside vs outside) differed markedly between continents. In Europe, *T. melanosporum* abundance outside the brûlé (63.8 ± 23.4 SE reads) showed an important 90.5% reduction, while in Australia, the decline was more moderate (408.2 ± 98.5 SE reads, 45.6% reduction), suggesting regional differences in how *T. melanosporum* colonizes areas beyond the visible brûlé zone. Site-level variation was modest (random effect variance = 0.21), indicating consistent patterns across plantations within each continent. The zero-inflation model showed that *T. melanosporum* presence probability was statistically higher inside the brûlé (0.91) compared to outside (0.31; *z* = 10.35, *P* < 0.001, Table S5), indicating that the fungus was frequently absent, as expected, from samples collected outside the brûlé zone (Fig. 5B) (Table S5). These results indicate that the brûlé primarily governs *T. melanosporum* occurrence, while continental differences emerge mainly in the strength of abundance suppression outside the brûlé.

Across all samples, *Tuber* was the most abundant fungal genus (Fig. 5C through E; also see Fig. S13 at https://doi.org/10.6084/m9.figshare.31329013) detected, accounting for a total relative sequence abundance of 10.3%. Other abundant soil fungi were *Fusarium* (8.3%), *Mortierella* (5.3%), *Cryptococcus* (5.0%), *Scleroderma* (3.3%), *Inocybe* (2.4%), *Hebeloma* (2.0%), and *Ilyonectria* (1.9%). *Tuber* was also the most abundant genus of all the 38 ectomycorrhizal genera (Fig. 5D) present in the data, with ~46% of relative sequence abundance, followed by *Scleroderma* (14.5%), *Inocybe* (10.3%), *Hebeloma* (8.9%), *Tomentella* (8.0%), *Trichophaea* (4.6%), *Tarzetta* (3.2%), *Russula* (0.9%), *Descolea* (0.8%), and *Entoloma* (0.7%). Regarding the relative sequence abundance of all the classified ectomycorrhizal species, *T. melanosporum* dominated with 43%, followed by other *Tuber* species, *T. rufum* f. *nitidum* (0.6%), *T. melosporum* (0.5%), and *T. oligospermum* (0.3%), which were only detected in Europe, and *T. brumale* (<0.1%) detected in Europe and eastern Australia (Fig. 5B and C)

**Fig 6:**
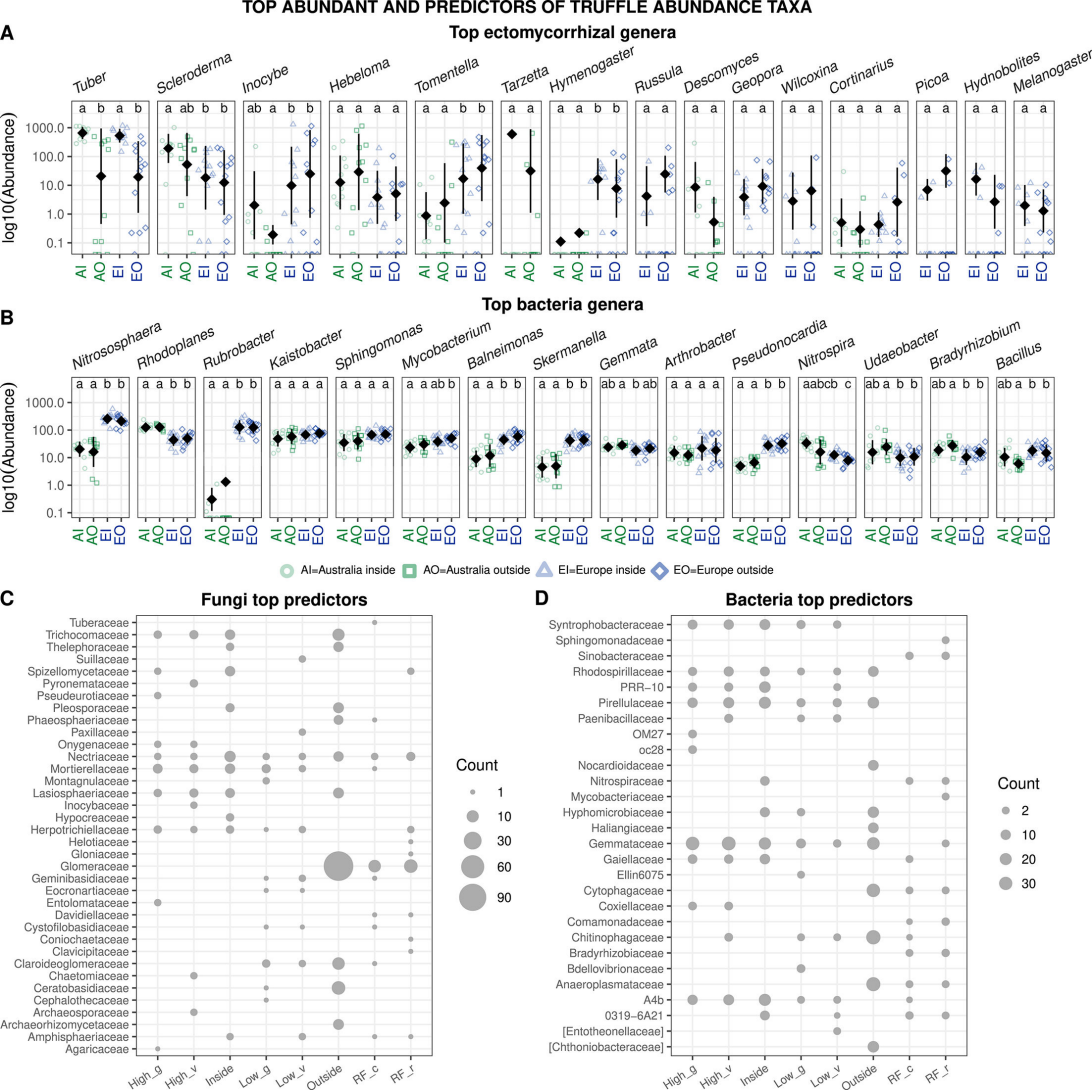
Differential abundance (in log_10_ scale) of the top ectomycorrhizal fungal (**A**) and top bacterial (**B**) genera across the continent and brûlé. = Candidatus *Nitrososphaera* and *Udaeobacter* = Candidatus *Udaeobacter*. Jittered points represent site averages, black diamonds represent the mean, and bars the standard deviation. Different letters represent significantly different pairwise Wilcoxon tests (*P*≤0.05) after Benjamini-Hochberg correction for each genera. Number of indicators and Random Forest selected fungal (**D**) and bacterial (**E**) predictors of *Tuber melanosporum* abundance (i.e. number of sequences) and high or low *T. melanosporum* levels (high, medium, low, as discretized sequence abundance using k means, see Fig. S12 at https://doi.org/10.6084/m9.figshare.31329013). High_g is the correlation to a high *T. melanosporum* level, High_v is the indicator value to a high *T. melanosporum* level, Inside is the correlation to the inside of the brûlé, Low_g is the correlation to a low *T. melanosporum* level, Low_v is the indicator value to a low *T. melanosporum* level, outside is the correlation to the outside of brûlé. RF_c is the number of random forest selected predictors using classification (i.e. *T. melanosporum* levels), RF_r is the number of random forest selected predictors using regression (i.e. *T. melanosporum* abundance).

**Fig 7:**
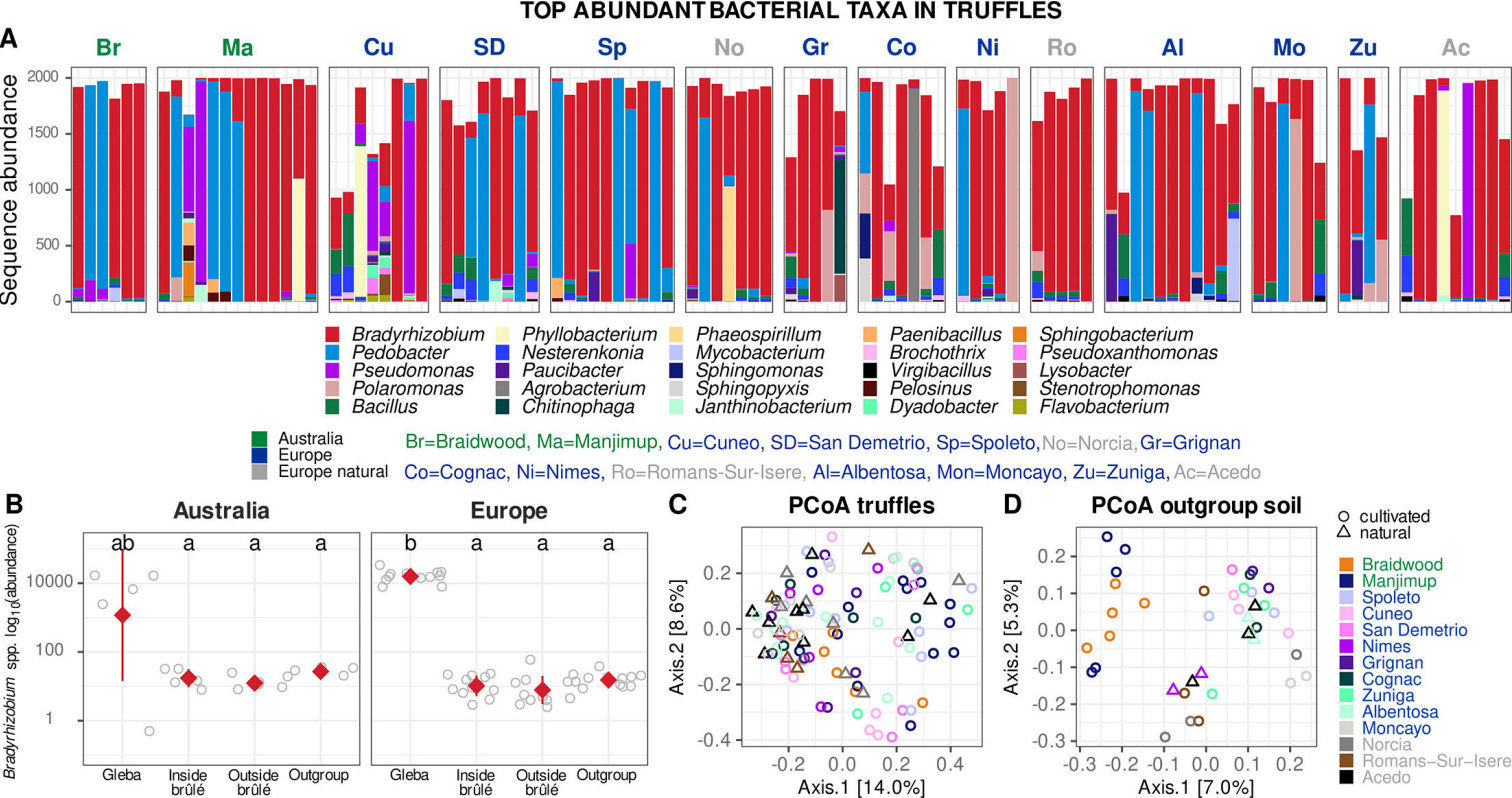
Rarefied sequence abundance of the top 25 bacterial genera in the truffle gleba (**A**), highlighting the enrichment of *Bradyrhizobium* in truffle gleba compared to the soil (**B**). Principal coordinate analysis (PCoA) based on Jaccard distance displaying the variability in the bacterial microbiome of truffles (**C**), and bacterial communities found in the soils where the truffles were collected (**D**). Data were previously rarefied (See M&M section for details). In B, y axis is in log_10_ scale, jittered points represent site averages, red diamonds represent the mean, and bars the standard deviation, while different letters represent significantly different pairwise Wilcoxon tests (*P* ≤ 0.05) after Benjamini-Hochberg correction.

Per-site averages across continent × brûlé levels comparisons allowed us to identify differences in main fungal and bacterial genera between Australian and European orchards (Fig. 6A through C). For instance, *Metarhizium*, *Ilyonectria,* and *Exophiala* were more abundant in the Australian orchards, while *Tetracladium*, *Hemathonectria, Phoma,* and *Acremonium* were more abundant in European orchards, respectively. Regarding the mycorrhizal genera, besides *Tuber* and *Scleroderma*, all the other genera that showed statistical differences between continents were more abundant in Europe compared to Australia (Fig. 6B). It is worth mentioning that some taxa, such as *Tarzetta* and *Descolea*, were found exclusively in Australian orchards. Indicator species analysis and random forest models found several OTU predictors of *T. melanosporum* sequence abundance within the brûlé (Fig. 7C). Regarding the fungi, Spizellomycetaceae, Mortierellaceae, Hypocreaceae, Herpotrichiellaceae, and Amphisphaeriaceae families contained the highest number of OTUs associated with *T. melanosporum* abundance. Mortierellaceae, Herpotrichiellaceae, and Amphisphaeriaceae were also the top random forest predictors of *T. melanosporum* sequence abundance (see M&M section for details on how *T. melanosporum* sequence abundance was discretized as well as Fig. S13). On the other end, Pleosporaceae, Nectriaceae, Glomeraceae, Claroideoglomeraceae, and Ceratobasidiaceae were the families with highest number of fungal predictors of low *T. melanosporum* sequence abundance.

The most abundant bacterial genera (Fig. 7C) *Nitrososphaera* (i.e. Candidatus *Nitrososphaera*), *Rubrobacter*, *Sphingomonas*, *Balneiomonas*, *Skermanella*, and *Pseudonocordia* were higher in European orchards compared to the Australian ones, while *Rhodoplanes*, *Nitrospira*, and *Bradyrhizobium,* but only outside the brûlé, were higher in the Australian orchards. We found that OTUs in the Syntrophobacteraceae, Rhodospirillaceae, Pirellulaceae, Gemmataceae, and A4b families were predictive of *T. melanosporum* and associated with a higher sequence abundance of *T. melanosporum* in soils within the brûlé. On the contrary, OTUs in the Xanthomonadaceae, Sphingomonadaceae, Sinobacteraceae, Cytophagaceae, Chitinophagaceae, and Anaeroplasmataceae were indicators of lower sequence abundance of *T. melanosporum* in the soil. Some families, such as Sinobacteraceae, Nitrospiraceae, Cytophagaceae, and Anaeroplasmataceae, contained OTUs that were important for random forest predictors of *T. melanosporum*.

### The microbiome of *T. melanosporum* truffles cultivated on different continents

The bacterial communities of *T. melanosporum* gleba were dominated by a single OTU belonging to *Bradyrhizobium* (54.2% relative sequence abundance on the total 141 genera), but also represented by *Pedobacter* (17.3%), *Pseudomonas* (4.2%), *Polaromonas* (3.4%), and *Bacillus* (2.5%). The top 25 abundant genera in the gleba (Fig. 7A) accounted for nearly 91% of the total sequence abundance. Four OTUs were classified as *Bradyrhizobium*. However, OTU41 was the dominant *Bradyrhizobium* OTU that comprised 99.98% of sequences that were conserved between the truffle gleba, soil, and outgroup soils. In the European orchards, *Bradyrhizobium* was significantly (*P* ≤ 0.05) enriched in the truffle gleba compared to soils (Fig. 7B). No clear biogeography patterns were visible in the PCoA of the truffle microbiomes (Fig. 7C), while the outgroup soil of the Australian orchard clearly separated from the European outgroup soils (Fig. 7D).

### Soil properties and their impact on *T*. *melanosporum* abundance and microbiome

**Fig 8:**
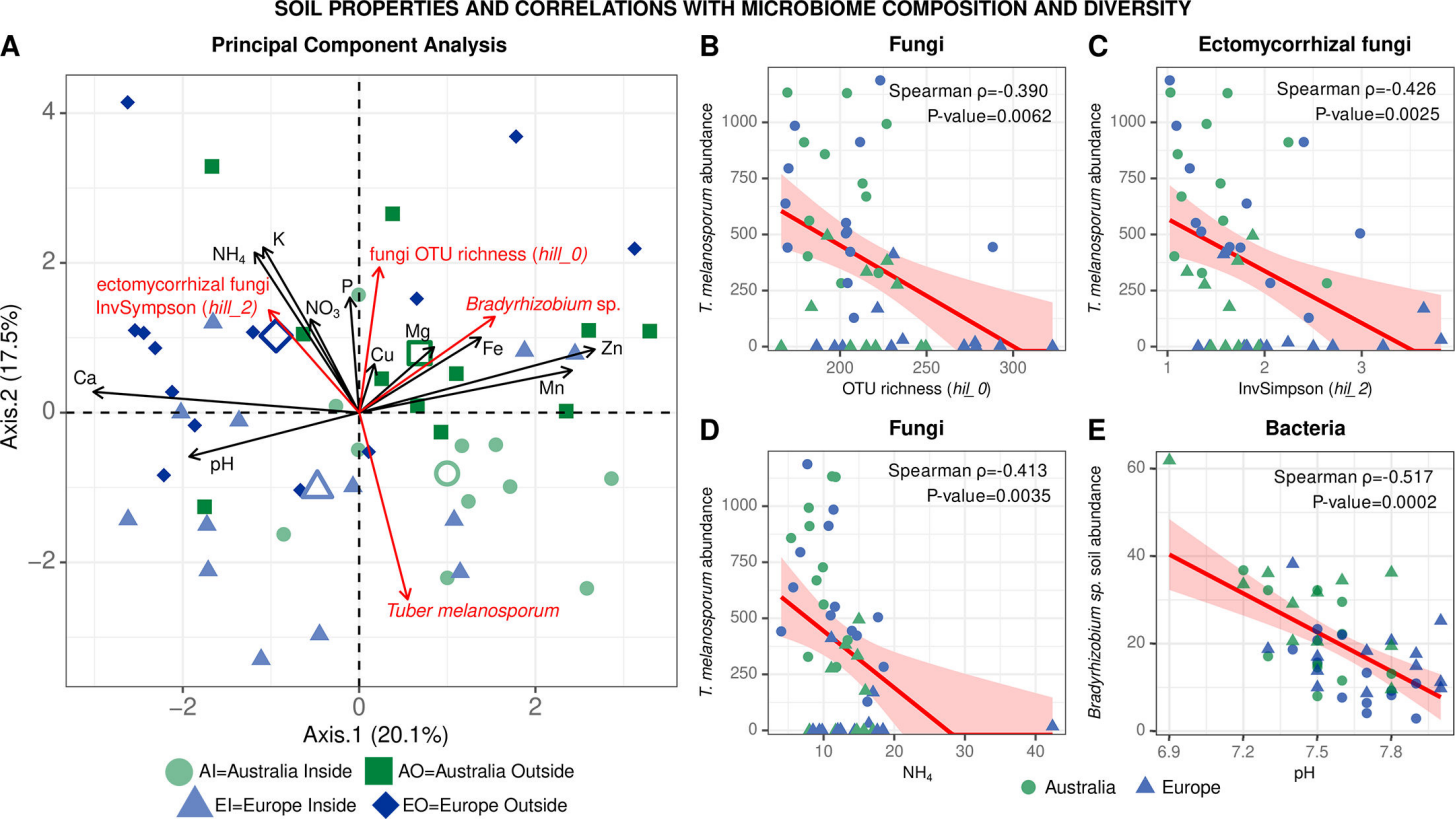
Principal component analysis of soil and microbiome properties (**A**). Open shapes represent group centroids, solid shapes represent individual soil and microbiome measurements; and scatter plots showing inverse relationships between *T. melanosporum* and OTU fungal richness (*hill_*0) (**B**); *T. melanosporum* and OTU ectomycorrhizal fungal inverse Simpson index (*hill_2*) (**C**); *T. melanosporum* and NH_4_ (**D**) and *Bradyrhizobium* soil abundance and pH (**E**). Significant Spearman correlation and *P* value after Benjamini-Hochberg correction are reported.

No significant (*P* ≤ 0.05) differences between Australia versus Europe and inside versus outside the brûlé were detected for any soil parameter measure in this study (see Tables S1 and S2; Fig. S14 at https://doi.org/10.6084/m9.figshare.31329013). Principal component analysis (Fig. 8A) clearly separates soils from Australia (right) and Europe (left) along the first axis. It also shows the trend of soil alkalinity with Ca/pH opposing Mn/Fe/Zn, which is common in calcareous soils. Axis 1 is structured by soil fertility N/P/K and separates observations from inside and outside the brûlé. It also shows that richness and InvSimpson are higher outside the brûlé, while *T. melanosporum* is higher inside. Significant Spearman correlations of −0.39 and −0.43 were found between *T. melanosporum* abundance and species richness or Inverse Simpson index, respectively (Fig. 8B and C). Further, we found that increasing NH_4_ significantly (*P* ≤ 0.05) and negatively impacts *T. melanosporum* abundance (Fig. 8D), while increasing pH significantly reduces the amount of *Bradyrhizobium* in the soil (Fig. 8E).

## DISCUSSION

Globally, the European black truffle species *T. melanosporum* is the most successful ectomycorrhizal fungus to be intentionally cultivated (20). First cultivated in its native range with artificially inoculated ectomycorrhizal tree seedlings in the 1970s, *T. melanosporum* is now being produced in North America, Australia, Asia, South America, and Africa (20). Australia is now the top *T. melanosporum* producer outside of Europe, which led to the hypothesis that *T. melanosporum* (and its host plant) may benefit from competition release in Australia.

In this study, we tested this hypothesis by characterizing soil fungal and bacterial communities inside and outside of the truffle brûlé in 24 productive truffle orchards across Australia and Europe. Continent effect on alpha diversity was mediated by a significant continent × brûlé interaction with significant continental differences in richness only for fungi outside brûlé, and Shannon diversity both inside and outside brûlé for fungi and outside for the ectomycorrhizal fungi. Few differences were detected for the bacteria as expected. European soils showed greater ectomycorrhizal diversity compared to Australian ones, which aligns with our hypothesis that the release of *T. melanosporum* from ectomycorrhizal competition in Australia leads to truffles becoming more dominant and over-represented in the ectomycorrhizal fungal community in truffle orchards across this continent; the reduced enemy pressure promotes *T. melanosporum* colonization outside the brûlé. Interestingly, European communities are more even, taxa contribute meaningfully to community structure and functionally or competitively relevant members, reflecting a more mature and stable microbiome. This was supported by the higher number of taxa we found in Europe compared to Australia, especially the ectomycorrhizal taxa outside of the brûlé.

*T. melanosporum* abundance was also impacted by the same continent × brûlé interaction with the Australian soil outside the brûlé having significantly higher truffle abundance than the European soil demonstrating its ability to colonize in reduced competition environments such as Australia. These results demonstrate differing ecological constraints on trufficulture and production in different regions of the world that are not often considered by truffle growers or scientists. We expect that competition release for *Tuber* is a common phenomenon in the Southern Hemisphere, given that *Tuber* evolved in the Northern Hemisphere with Northern Hemisphere plant lineages, but additional studies in other Southern continents will be needed to confirm this hypothesis.

### Fungal community diversity in European and Australian truffle orchards

Fungal communities are known to be geographically structured, due to dispersal limitation, among other factors (66, 67). Hence, similarity between fungal communities at different sites tends to decay as the distance between sites increases (68). This pattern of decay by distance was demonstrated here with Jaccard and Bray-Curtis dissimilarity between fungal communities between each continent. In fact, geography was the greatest explanatory factor of fungal community similarities in distance-based permutational multivariate analysis of variance. The pattern of decay by distance was further recapitulated at a continental scale in both Europe and Australia and showed geographic structuring of the fungal communities at continental scales.

*T. melanosporum* clearly was thriving across these sites, as it accounted for the most abundant sequence within this study. The brûlé is a characteristic feature produced by black truffle mycelium that reduces vegetation and impacts to the soil microbial communities (28). Although the brûlé did not necessarily impact the richness of fungi in this zone, the fungal community structure differed between the brûlé and the surrounding soil. In fact, the brûlé was the most important factor for the distance-based permutational multivariate analysis of variance (PERMANOVA) of the ectomycorrhizal community, while much less impactful for the total fungi and the bacteria. Other ectomycorrhizal genera were abundant across our study sites, including *Scleroderma*, *Hebeloma*, and *Inocybe* (particularly in Europe). *Scleroderma* can be common in truffle orchards, particularly young orchards where its fruiting may precede that of truffles (69, 70). However, it is still not clear whether *Scleroderma* and *Tuber* compete directly, or if they have somewhat distinct ectomycorrhizal niches (71). Studies on the ectomycorrhizal diversity of truffle plantations in Europe highlight the great diversity of species and genera of Ascomycetes and Basidiomycetes that coexist and compete with truffles in plantations (De Miguel et al., 2014). The presence of Thelephorales is particularly noteworthy and is one group that we found at a greater abundance in Europe compared to Australia. Although *Tarzetta* is a Northern Hemisphere ectomycorrhizal genus, it was only detected from Australian sites, another indication of how the competitive landscape changes when species are introduced to new geographic regions. Interestingly, *Descolea*, an ectomycorrhizal genus native to Australia often associated with eucalyptus, was detected in Australian orchards indicating that this Australian taxon is likely able to jump hosts and associate with oak (72). While it is evident from our sequence data that many non-Australian ectomycorrhizal fungal taxa have been introduced into Australia, it is important to note that we did not detect any other *Tuber* species in Australia aside from *T. melanosporum* and *T. brumale. T. brumale* is known to have accidentally been introduced into Eastern Australia, New Zealand, and the United States (73–75).

### Bacterial community diversity in European and Australian truffle orchards

Bacterial diversity has been shown to have biogeographic patterns that are largely shaped by edaphic factors such as pH (41). While pH was a relatively controlled variable in the soils that we studied, given that acid soils in Australia were limed to increase soil pH agronomically, the bacterial communities in European and Australian soils were very different from each other in composition. Continent was the greatest explanatory factor of bacterial communities in our distance-based permutational multivariate analysis of variance. Yet bacterial community richness was quite similar in magnitude, both inside and outside of the brûlé. In contrast to what has been found previously, within each continent, there was surprisingly little evidence of strong patterns in bacterial beta diversity in truffle orchards, either between sites or inside or outside the brûlé (28). This indicates that the bacterial community at large is unlikely to limit truffle productivity or to account for differences in the abundance of *T. melanosporum* between sites, regions, or continents.

### Fungal and bacterial predictors of *T. melanosporum* abundance

It has been shown that the concentration of *T. melanosporum* in soil is a strong indicator of the potential for truffle ascocarp production in subsequent years (76). It is likely that there are soil microbes that may facilitate or inhibit *T. melanosporum* growth, either directly or indirectly, but so far, these have yet to be confirmed (77, 78). Given our expansive data set, which included over 50 million sequences across 522 samples, we used both indicator species analysis and random forest modeling to try to identify putative truffle helper microbes. Interestingly, Mortierellaceae, Herpotrichiellaceae, and Amphisphaeriaceae were the top predictors of *T. melanosporum* sequence abundance. They were also identified as significant indicator taxa. Mortierellaceae are common soil fungi, and we found them to be among the most abundant fungi within brûlés. Mortierellaceae have previously been associated with productive truffle sites (77, 78). Some species of Mortierellaceae are plant growth promoters (79). These fungi have chitinolytic abilities and are thought to be important in the recycling of dead fungal biomass, thereby increasing the availability of N that would otherwise be locked up in this biopolymer (80). It is unclear what roles Herpotrichiellaceae and Amphisphaeriaceae species may play in truffle orchard soils, but they may indirectly impact *T. melanosporum* by preying on other fungi or plants growing in the zone of the brûlé. For instance, some species of Herpotrichiellaceae are myco-parasites, whereas some species of Amphisphaeriaceae are known plant pathogens (81, 82). In contrast, arbuscular mycorrhizal fungi (e.g., Glomeraceae and Claroideoglomeraceae) were among those fungi predictive of low *T. melanosporum* abundance. This may be an indirect indicator, perhaps indicative of non-host vegetation, which would compete with truffles for host tree water and nutrients. While bacterial OTUs in Syntrophobacteraceae, Rhodospirillaceae, Pirellulaceae, and Gemmataceae were predictive of higher *T. melanosporum* abundance, it is not clear what specific functions or interactions the bacteria may have in this ecosystem (81, 82).

### The truffle microbiome

Truffles are known to host their own microbiome, which is thought to be important in truffle aroma, metabolism, and possibly defense (2, 83). There are questions as to how stable in structure the truffle microbiome is, and whether it may change when cultivated in different soils having very different bacterial communities (84). We hypothesized that the bacterial microbiome of *T. melanosporum* would be well conserved between European and Australian cultivated truffles given that previous research across different soils in Europe reported a consistent core truffle microbiome. Indeed, supporting our hypothesis and perhaps surprisingly, we found no clear biogeographic patterns in the truffle microbiome, despite strong differences in soil bacterial communities between continents. Of the 141 bacterial taxa detected as part of the truffle microbiome, *Bradyrhizobium, Pedobacter, Pseudomonas*, and *Polaromonas* were particularly abundant and common. However, a single OTU belonging to *Bradyrhizobium*, previously reported from the truffle microbiome, dominated the truffle microbiome across all sites sampled (34, 36). Questions may arise as to the origin of this bacterium, and whether it was introduced to the system through the inoculation of seedlings with truffle sporocarps, the common commercial approach for establishing truffle seedlings for planting in truffle orchards.

Across multiple studies on different *Tuber* species, *Bradyrhizobium* has repeatedly been detected as one of the most abundant bacterial taxa inside truffle fruiting bodies (34, 78, 85, 86). However, hypotheses regarding the role of *Bradyrhizobium* in truffles remain controversial. Some *Bradyrhizobium* taxa are known to convert atmospheric N₂ into bioavailable nitrogen. In 2010, Barbieri et al. showed that genes homologous to nif clusters are present in *Tuber*-associated *Bradyrhizobium*, and that some isolates exhibit nitrogenase activity *in vitro* (87). More recently, Graziosi and team isolated *Bradyrhizobium* from *Tuber magnatum* ascomata and were able to directly amplify nifH genes from the bacterial isolate (88). Therefore, it is hypothesized that N-fixation by *Bradyrhizobium* may drive protein synthesis and growth that occur during *Tuber* ascocarp development, which may be a particularly important function given that truffles often form in nutrient-poor soils. However, despite the overwhelming evidence that *Bradyrhizobium* is enriched within truffle fruiting bodies, it is still not clear whether, or how much N-fixation actually occurs within truffles (36). Aside from N-fixation, *Bradyrhizobium* may have other key functions that justify its enrichment, such as the production of auxin-like compounds (e.g., IAA), vitamins (e.g., biotin and riboflavin), and siderophores (i.e., iron chelators), which may act as growth factors or signaling molecules influencing *Tuber* growth and maturation. Another important phenomenon that may benefit this fungal–bacterial association is the potential contribution of *Bradyrhizobium* to microbial exclusion (via competition, nutrient sequestration, or antimicrobial activity), thereby helping stabilize a “beneficial” truffle core microbiome and reduce pathogen load.

Our sampling of soils outside of the Australian orchards that were not limed, amended, or planted with truffle-inoculated seedlings shows a low abundance of the most dominant *Bradyrhizobium* OTU in truffle ascocarps, indicating the bacterium likely naturally exists in Australian soils and was not introduced during the inoculation process. Rather, it appears that *T. melanosporum* may select *Bradyrhizobium* and other core microbiome members from the diverse microbiota present in its soil environment. Further, *Bradyrhizobium* was not dominant or present in every truffle sampled, meaning this bacterium is not essential for *T. melanosporum* to fruit or mature. Rather, the truffle bacterial microbiome shows variation in structure, even within sites, which may vary with age, condition, environment, and has the potential to impact truffle aroma, health, and post-harvest longevity.

### Conclusions

In conclusion, the release of *T. melanosporum* to ectomycorrhizal competition in Australia is consistent with our findings providing new insights into the ecology and biology of truffles and their related microbiomes. This intercontinental study is the first to provide a comprehensive comparison of truffle soil microbiome from European areas where *T. melanosporum* is autochthonous and other areas of the world where this species has been introduced. Our results demonstrate for the first time how reduced ectomycorrhizal diversity and competition in Australian truffle orchards favors the abundance of *T. melanosporum* in the soil community, reiterating the importance of establishing truffle plantations in soils that are potentially free of ectomycorrhizal and even microbial competition. We also confirmed the impact of the brûlé produced by *T. melanosporum* on the diversity and composition of EcM communities, while finding much lower effects on bacterial communities. The fungal families Mortierellaceae, Herpotrichiellaceae, and Amphisphaeriaceae and the bacterial families Syntrophobacteraceae, Rhodospirillaceae, Pirellulaceae, Gemmataceae, and A4b were found to be predictors of high *T. melanosporum* abundance. Finally, the study confirmed the dominant role of *Bradyrhizobium* in the truffle sporocarp microbiome both in Europe and Australia, although its function and importance for the truffle is still uncertain.

## Data Availability

Raw ITS and 16S rRNA sequence data (.fastq files) have been deposited in the NCBI Sequence Read Archive (SRA) database (89) under BioProject accession PRJNA1279640. All scripts and R code required to reproduce the analyses are publicly available on GitHub at https://github.com/Gian77/Published-R-Code/tree/master/Benucci_etal_2025_CompetitionReleaseTruffle. Supplementary materials are archived on Figshare (https://doi.org/10.6084/m9.figshare.31329013). Minor adjustments to figure labels were performed using Inkscape (90).
